# Microbiological hazard analysis of ready-to-eat meats processed at a food plant in Trinidad, West Indies

**DOI:** 10.3402/iee.v3i0.20450

**Published:** 2013-07-19

**Authors:** Stacey-Marie Syne, Adash Ramsubhag, Abiodun A. Adesiyun

**Affiliations:** 1 Department of Life Sciences, The University of the West Indies, St. Augustine, Trinidad; 2 School of Veterinary Medicine, The University of the West Indies, St. Augustine, Trinidad

**Keywords:** contamination, zoonotic pathogens, processed meat, processing

## Abstract

**Background:**

A bacteriological assessment of the environment and food products at different stages of processing was conducted during the manufacture of ready-to-eat (RTE) chicken franks, chicken bologna and bacon at a large meat processing plant in Trinidad, West Indies.

**Methods:**

Samples of air, surfaces (swabs), raw materials, and in-process and finished food products were collected during two separate visits for each product type and subjected to qualitative or quantitative analysis for bacterial zoonotic pathogens and fecal indicator organisms.

**Results:**

*Staphylococcus aureus* was the most common pathogen detected in pre-cooked products (mean counts = 0.66, 1.98, and 1.95 log_10_CFU/g for franks, bologna, and bacon, respectively). This pathogen was also found in unacceptable levels in 4 (16.7%) of 24 post-cooked samples. Fifty percent (10 of 20) of pre-cooked mixtures of bacon and bologna were contaminated with *Listeria* spp., including four with *L. monocytogenes*. Pre-cooked mixtures of franks and bologna also contained E. coli (35 and 0.72 log_10_ CFU/g, respectively) while 5 (12.5%) of 40 pre-cooked mixtures of chicken franks had *Salmonella* spp. Aerobic bacteria exceeded acceptable international standards in 46 (82.1%) of 56 pre-cooked and 6 (16.7%) of 36 post-cooked samples. Both pre-and post-cooking air and surfaces had relatively high levels of aerobic bacteria, *Staphylococcus aureus* and coliforms, including equipment and gloves of employees. A drastic decrease in aerobic counts and *Staphylococcus aureus* levels following heat treatment and subsequent increase in counts of these bacteria are suggestive of post-cooking contamination.

**Conclusion:**

A relatively high level of risk exists for microbial contamination of RTE meats at the food plant investigated and there is a need for enhancing the quality assurance programs to ensure the safety of consumers of products manufactured at this plant.

Contamination by pathogenic microorganisms is one of the most important challenges faced by producers of processed meat products. The presence of foodborne pathogens in meat and meat products can result in a range of human health problems as well as economic losses to producers due to recalls from market places (1). Ready-to-eat (RTE) meats are especially a concern since these may be consumed without further cooking and are known to be good growth substrates for pathogenic microorganisms such as *Listeria monocytogenes* (2). Contamination of RTE meats by pathogenic bacteria has been previously reported in Trinidad. A voluntary recall by one manufacturer in Trinidad in 2003 was due to contamination of *L. monocytogenes*, but other organisms were detected in finished meat products at the plant, including *E. coli*, *Salmonella* spp., *Campylobacter* spp., and unacceptable levels of aerobic bacteria (3). Additionally, Hosein et al. (4) detected *L. monocytogenes* and *E. coli* in deli meat samples (1.4% and 2.9%, respectively) collected from local supermarkets on the island. The occurrence of pathogenic microorganisms in RTE meats in Trinidad indicates the need for improved quality assurance by local producers in order to reduce consumers’ risks of exposure to infectious foodborne agents.

Ensuring good quality raw materials, adequate lethality treatment, and effective sanitation of both the equipment and processing environment are crucial in preventing contamination of RTE meats. The presence of pathogens on surfaces of equipment or the environment, particularly in post-cooking areas, serves as one of the most important routes for contamination of RTE meats (2). Some of these organisms, such as *Listeria* spp., can be present in raw meat, which may serve as a source for cross-contamination of finished products (5). Contamination of processed foods can also occur due to poor quality water and unacceptable levels of airborne microorganisms in the processing environment (6, 7). Furthermore, temperature and humidity in the food-processing environment play an integral role in the quality of products manufactured (8). The use of low ambient temperatures during production diminishes the ability of microorganisms to grow and replicate. However, lowering temperature can also result in increasing relative humidity, and both of these factors are known to favor the development of bacterial growth (9).

Several microbiological guidelines for processed foods have been established by agencies, including the World Health Organization and the United States Food and Drug Administration. These guidelines are commonly used for developing food safety programs such as Hazard Analysis and Critical Control Points (HACCP), Good Manufacturing Practices (GMPs), and Good Hygienic Practices (GHPs) (10).

In Trinidad and Tobago, the Chemistry, Food and Drug Division of the Ministry of Health is the main authoritative body, which issues certificates for sanitation and the sale of goods for local and international markets. The Division also provides food inspection and laboratory services, but rely on FAO food quality standards due to a lack of local legislative guidelines (11). Despite their presence, manufacturers frequently do not adhere to food safety standards and there is a vital need for upgrading and improving local food safety legislation as well as its enforcement (11).

The problem of contamination of RTE meats produced in Trinidad has been documented. However, there is no data available on plant factors which may be influencing the quality of finished products. The objective of this study was to assess the effectiveness of microbiological control at critical points during processing operations at one plant manufacturing RTE meats in Trinidad, West Indies. The plant is one of the largest on the island and was selected based on the results of a survey that showed the presence of pathogenic and indicator species of bacteria in retail RTE products manufactured by the facility (11).

## Materials and methods

### Sampling

Investigations were carried out over the period June–November, 2007, during the production of chicken franks, chicken bologna and bacon. Microbial analysis was conducted on air, pre-processed and post-processed food, and surfaces, including equipment, floors, packaging material, and workers’ apparels at each process step. Duplicate samples were collected on six occasions (twice for each product) over the study period. Information on the plant design and layout as well as the processing operations was obtained from observations made during the visits and from a questionnaire completed by interviewing the plant manager. The number of samples (*n*) collected for each product or environment was determined using the formula *n*=*z* ^2^ (*p*) (1 – *p*)/*d* ^2^ (12), where *z*=1.96; *p*=prevalence rates from previous studies (3, 13); and *d*=0.21. A total of 24 samples each of raw meat, uncooked formulated products, cooked products, and air in addition to 48 raw non-meat ingredients and 50 swabs of surfaces were subjected to microbiological analysis. Samples of food, water, and ingredients were aseptically collected in sterile glass bottles or stomacher bags (500 ml) and stored on ice before processing in the laboratory within 24 hours of collection. Surfaces were swabbed and air quality was evaluated using the impaction or filtration methods. Relative humidity and temperature of each processing area were measured using data loggers (HOBO^®^ H8 Pro RH/Temperature Logger, Bourne, MA, USA).

### Sample processing

#### Raw materials and food

Raw materials and food were analyzed following previously described methods (14–18) using Oxoid culture media and supplements (Oxoid Ltd., Cambridge, UK). Samples (25 g) were homogenized in 225 ml lactose broth (LB) using a stomacher (Seward Stomacher 400 Lab System, West Sussex, UK) for 2 min at high speed. The homogenate was then serially diluted and spread-plated (0.1 ml) on a range of enumeration agar plates: MacConkey agar (MAC) for coliforms; eosin methylene blue (EMB) for *E. coli*; sorbitol MacConkey agar (SMAC) for *E. coli* O157; mannitol salt agar (MSA) for *S. aureus*; plate count agar (PCA) for heterotrophic bacteria (total aerobic plate counts). The agar plates were incubated (Thermo Fisher Scientific Inc., Waltham, MA, USA) at 37°C for 24 hours after which colonies were counted using a Quebec Dark field colony counter (Cambridge Instruments Inc., Buffalo, NY, USA). When plate exceeded 300 colonies, further serial dilutions and plating were immediately carried out (within 1–3 hours) on dilutions stored in a refrigerator (Jordan Commercial Refrigerator Co., Philadelphia, PA, USA).

For detection of *Salmonella* spp., the remaining homogenized mixture from the stomacher bag was incubated for 24 hours at 37°C for pre-enrichment of the organism. Following enrichment in Selenite Cystine (SC) and Tetrathionate (TT) broths, presumptive *Salmonella* spp. was detected by streaking on xylose lysine desoxycholate (XLD) agar, brilliant green agar (BGA) and bismuth sulfite agar (BSA).

*Listeria* spp. was detected by stomaching 25 g of product with in 225 ml *Listeria* enrichment broth (LEB) before incubating at 30°C for 48 hours. The LEB cultures were then streaked on *Listeria* selective agar (LSA) plates that were incubated at 37°C for 24 hours.

Representative colonies from plates (except PCA) were purified and streaked on blood agar (BA) plates before verification of identity using standard biochemical methods (14–18), including for *E. coli* and *Salmonella* spp. – growth and reaction on triple sugar iron agar and lysine iron agar; indole–methyl red–Vogues Proskauer–citrate (IMViC) and urease tests; for *S. aureus* – coagulase test; and for *Listeria* spp. – growth and reaction on bile esculine agar and motility medium; methyl red–Vogues Proskauer (MRVP), sulfur indole motility and urease tests. Additionally, presumptive *Salmonella* isolates were subjected to β-galactosidase test using ONPG discs (Oxoid Ltd., Cambridge, UK) and slide agglutination tests with *Listeria* polyvalent antiserum, one and four (Difco, Michigan, USA). Cultures of presumptive *Salmonella* spp. were also sent to the regional reference laboratory (Caribbean Epidemiology Center, Port of Spain, Trinidad) for further serological confirmation of identity.

#### Sampling of water, surfaces, and air

The general methods described for processing raw materials and foods above were also applied to water, swabs, and air samples with the following variations. Water samples were spread plated (0.1 ml) onto EMB, MAC, SMAC, MSA, and PCA agar plates with and without dilutions. Additional samples (100 ml) of water were filtered (sterile 0.45 µm membranes; Millipore, Billerica, MA, USA) for enumeration of total coliforms and fecal coliforms using mEndo and mFC agar (Difco Laboratories Inc., Michigan, USA), respectively (19). Samples (100 ml) were also filtered and entire membranes were incubated in 9 ml LB or LEB for detecting *Salmonella* spp. and *Listeria* spp., respectively, as described earlier. Swabs (∼300 cm^2^) of equipment, employees’ gloves (both right and left) and coats, and floors were placed in 5 ml sterile saline and vortexed before plating on enumerative agar. Additional swabs were incubated in 9 ml LB and LEB for *Salmonella* spp. and *Listeria* spp.

Air was sampled by impaction directly on enumeration agar plates at a flow rate of 28 L/min (100 L for PCA and 200 L for all other media) using the SKC Biostage Impactor (SKC, PA, USA) (20). For detection of *Salmonella* spp. and *Listeria* spp., air (200 L) was filtered through 0.45 µm nitrocellulose membranes (Nalgene Analytical Test filters, Rochester, NY, USA) at a flow rate of 8 L/min. The filters were then incubated in 9 ml of LB and LEB and analyzed as described earlier. Microbial evaluation of bacon packages was done by thoroughly rinsing with sterile saline (20 ml), which was processed similarly to the water samples.

#### Statistical analyses

Analyses of the resultant bacterial counts were done with the use of Statistical Package for Social Sciences (SPSS) Version 15.0. The data were subjected to non-parametric Chi-square tests in addition to a Mann–Whitney U test and Kruskal–Wallis one-way analysis of variance (21).

## Results

### Plant layout and food safety programs

**Fig. 1:**
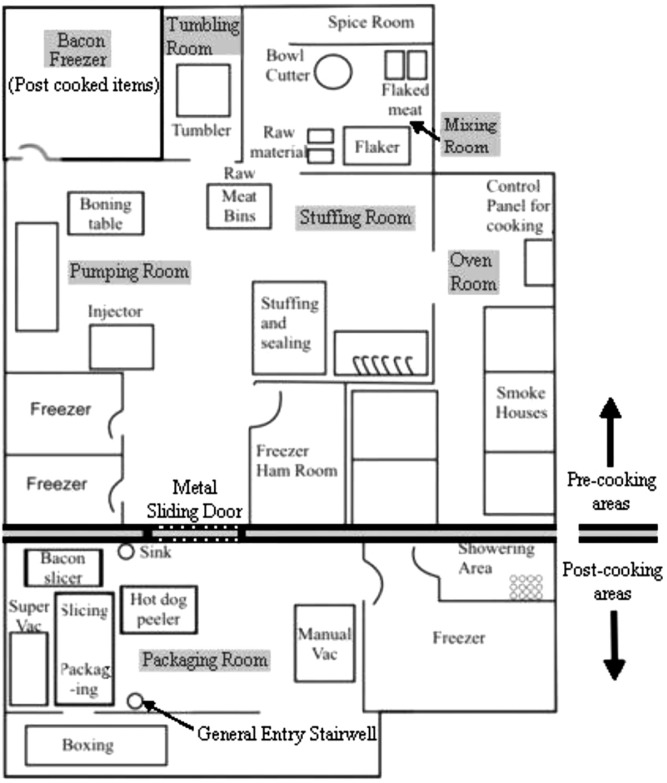
Sketch map of production floor and various processing rooms of plant manufacturing chicken franks, chicken bologna, and bacon.

During the manufacture of the products under study, seven different rooms were used (Table 1; Fig. 1). A metal sliding door separated pre-cooking from post-cooking areas and was kept ajar for varying periods of times during the day to facilitate employee and product traffic. One freezer in the pre-cooking area was designated to hold post-cooked bacon. The general entrance and exit for employees in both pre- and post-cooking areas was the stairwell located in the packing room. Regarding the plant's HACCP program, the only CCP identified was the cooking step in the oven room. Cooking times for chicken franks, bologna and bacon were 1.5, 6 and 3.5 hours, respectively and the oven temperature for all products was 78°C. Raw materials were used on a First In/First Out basis ensuring that ingredients did not stay in storage long enough to harbor high microbial counts and final products were tested using both in-house laboratories as well as regular validation from external laboratories. Regarding storage temperatures, raw meats requiring storage in cold temperatures were adequately stockpiled in freezers with temperatures ranging from 0 to −20°C and post-cooked products were housed in refrigerators set between 2 and 4°C to maintain shelf-life. Footbaths, external auditing of their HACCP program and frequent adenosine triphosphate (ATP) testing of equipment were all strategically implemented by this processing plant in an effort to decrease the incidence of cross-contamination. Employees were also given mandatory briefings on GHPs and GMPs before hire and the manufacturer posted reminders of these in the plant to help maintain sanitation measures.

**Table 1:** Stages during the manufacture of chicken franks, bologna, and bacon

Product	Room name	Category	Manufacturing process
Chicken Franks and Bologna	Mixing room	Pre-cooking	Raw meat flaked and mixed with filler to form an emulsion
	Stuffing room	Pre-cooking	Emulsion placed in sausage casing
	Oven room	Pre-cooking	Heat treatment applied
	Packaging room	Post-cooking	Peeling, slicing and vacuum packaging
Bacon	Pumping room	Pre-cooking	Raw meat pumped with cure
	Tumbling room	Pre-cooking	Pumped meat massaged in the tumbler
	Oven room	Pre-cooking	Cured meat smoked and cooked
	Bacon freezer	Post-cooking	Smoked meat left to freeze
	Packaging room	Post-cooking	Slicing and vacuum packaging

### Aerobic bacteria in food, air, water and environmental samples

During the manufacture of chicken franks, chicken bologna and bacon, the frequency of pre-cooked food samples exceeding acceptable limits of aerobic bacteria (10^5^ CFU/g) were 88.9% (16/18), 88.9% (16/18), and 70.0% (14/20), respectively, and for post-cooked samples, 25.0% (2/8), 0.0% (0/8) and 50% (4/8), respectively and this difference was significant (*P*<0.05). A total of 52 (65.0%) of 80 food samples exceeded acceptable limits, with the majority of post-cooked samples being detected during bacon manufacture (four samples).

**Fig. 2:**
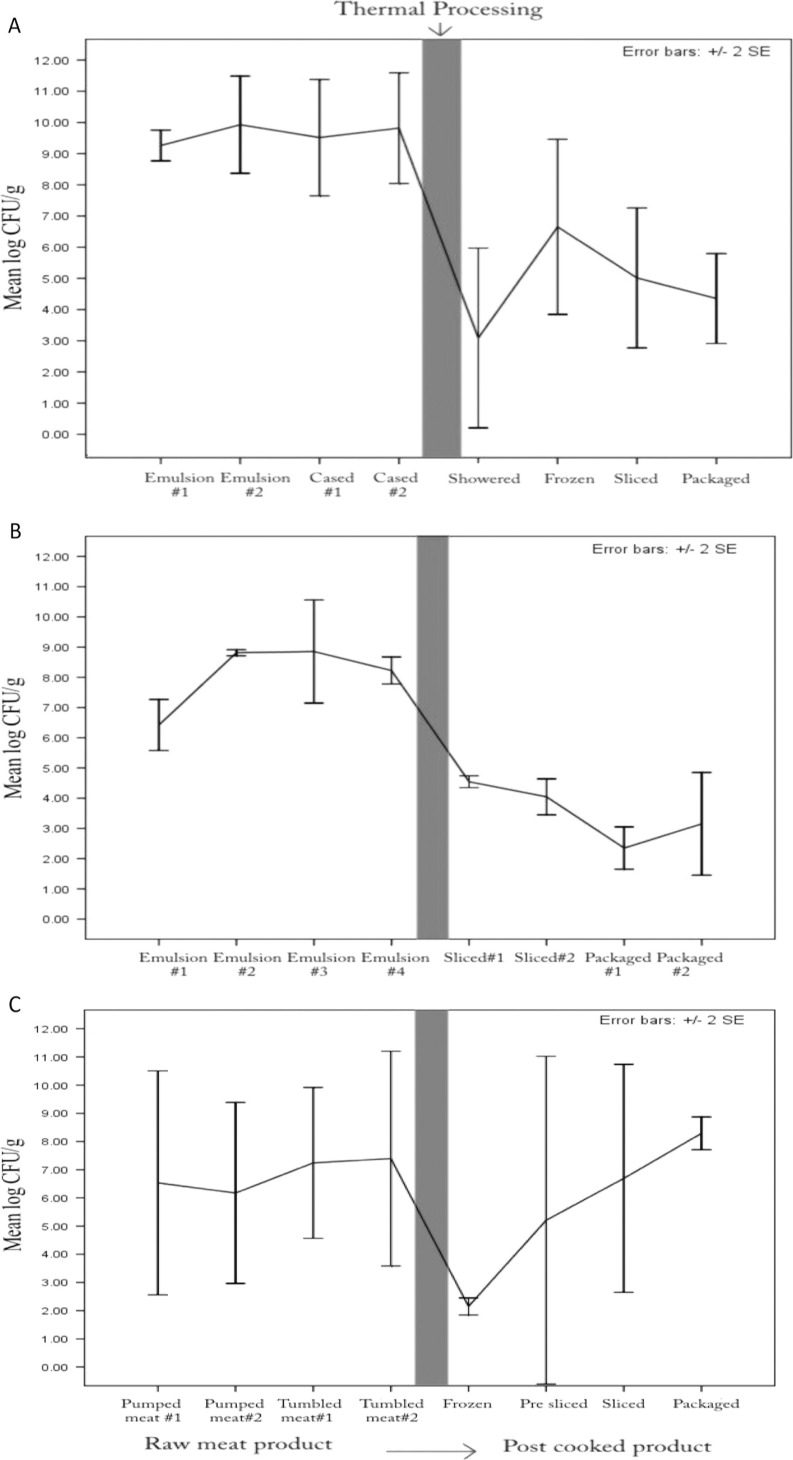
Total aerobic plate counts of pre- and post-cooking samples during production of chicken franks (A), chicken bologna (B), and bacon (C).

Average aerobic plate counts of franks and bologna were significantly lower (*P*<0.05) in post-cooked food samples (3.63 and 3.52 log_10_CFU/g, respectively) than in those that were pre-cooked (8.45 and 8.02 log_10_CFU/g, respectively) (Table 2). However, during bacon production, there was no significant difference (*P*>0.05) between aerobic counts in pre-cooked food samples (mean = 6.66 log_10_CFU/g) and post-cooked samples (mean = 5.59 log_10_CFU/g). Heat treatment resulted in a drastic decrease in TAPC per gram for all three products, but bacterial levels in bacon gradually increased in successive steps after thermal processing (Fig. 2).

**Table 2:** Counts of bacteria in air, surfaces, food, and water at plant producing chicken franks, chicken bologna, and bacon

		Air			Water			Surfaces			Food		
					
Bacterialspecies/group	Product	Pre-cook (log_10_CFU/m^3^)±SE	Post-cook (log_10_CFU/m^3^)±SE	*P*	Pre-cook (log_10_CFU/ml)±SE	Post-cook (log_10_CFU/ml)±SE	*P*	Pre-cook (log_10_CFU/m^2^)±SE	Post-cook (log_10_CFU/m^2^)±SE	*P*	Pre-cook (log_10_CFU/g)±SE	Post-cook (log_10_CFU/g)±SE	*P*
*E. coli*	Franks	–a	–	na^c^	–	–	na	–	–	na	0.35±0.35	–	NS^d^
	Bologna	–	–	na	–	–	na	0.81±0.81	–	NS	0.72±0.49	–	NS
	Bacon	–	–	na	No sample^b^	–	na	0.96±0.96	–	NS	–	–	na
Coliforms	Franks	1.35±0.46	1.05±0.35	NS	–	–	na	8.57±0.39	6.67±1.59	NS	7.09±0.69	0.58±0.38	<0.001
	Bologna	0.54±0.34	0.85±0.85	NS	–	–	na	9.17±0.34	5.08±2.28	NS	5.59±0.69	0.42±0.42	<0.001
	Bacon	–	–	na	No sample	–	na	7.06±1.03	6.01±0.78	NS	3.01±0.68	–	0.025
*E. coli* O157	Franks Bologna Bacon	– – –	– – –	ns na na	– – No sample	– – –	na na na	– – –	– – –	na na na	– – –	– – –	na na na
*S. aureus*	Franks Bologna Bacon	0.92±0.42 1.19±0.39 2.24±0.02	0.92±0.92 1.27±0.27 0.65±0.65	NS NS NS	– – No sample	– – –	na na na	0.69±0.47 2.42±0.88 0.83±0.83	– 2.23±1.41 1.45±0.92	NS NS NS	0.66±0.46 1.98±0.68 1.95±0.57	– 1.63±0.79 0.25±0.25	NS NS NS
Aerobic bacteria (TAPC)	Franks Bologna Bacon	2.62±0.13 2.78±0.09 2.08±0.17	2.33±0.15 2.19±0.15 1.72±0.24	NS NS NS	0.80±0.80 1.62±0.62 No sample	0.33±0.33 2.94±0.15 2.05±0.51	NS NS na	9.25±0.43 9.68±0.35 8.93±0.32	10.67±0.36 7.56±1.68 9.32±0.52	0.013 NS NS	8.45±0.55 8.02±0.49 6.66±0.56	3.63±0.77 3.52±0.37 5.59±1.09	<0.001 <0.001 NS

a No bacterial counts were observed.

b No sample was available.

c na, not applicable; no value could have been calculated.

d NS, not significant (*P*>0.05).

Mean aerobic counts of air samples ranged from 1.72 to 2.78 log_10_CFU/m^3^ (Table 2). There was no significant (*P*>0.05) difference in counts between pre-cooking and post-cooking areas. However, there was a general trend of decreasing TAPCs in air from early stages of processing (mixing room) to later stages (packaging room), especially during the manufacture of franks (Table 3). Swab counts of aerobic bacteria in post-cooking areas were significantly (*P*<0.05) higher than pre-cooking areas during the production of franks (Table 2). However, no significant differences were observed during the production of bacon and bologna.

**Table 3:** Mean total aerobic plate counts of air in various processing rooms

	TAPC (CFU/m^3^)		
	
Room	Franks	Bologna	Bacon
Mixing	830±180	645±405	**–** a
Stuffing	450±110	725±75	255±95
Tumbling	–	–	55±35
Oven	230±100	605±45	175±15
Packaging	225±75	165±55	60±30

a Room not used during processing.

Heterotrophic bacteria were detected in water at pre-cooking and post-cooking areas during processing of all three products. However, no significant differences were observed between samples taken at either stage (Table 2). However, three samples had heterotrophic counts that exceeded the US Environmental Protection Agency (USEPA) drinking water specification of 500 CFU/ml; and thus did not conform to guidelines established by the US Food Safety Inspection Service (22). These samples were taken from water used for showering post-cooked meats: two during bologna production (940 and 1,080 CFU/ml) and one during bacon production (1,350 CFU/ml).

### Prevalence of *S. aureus*

**Fig. 3:**
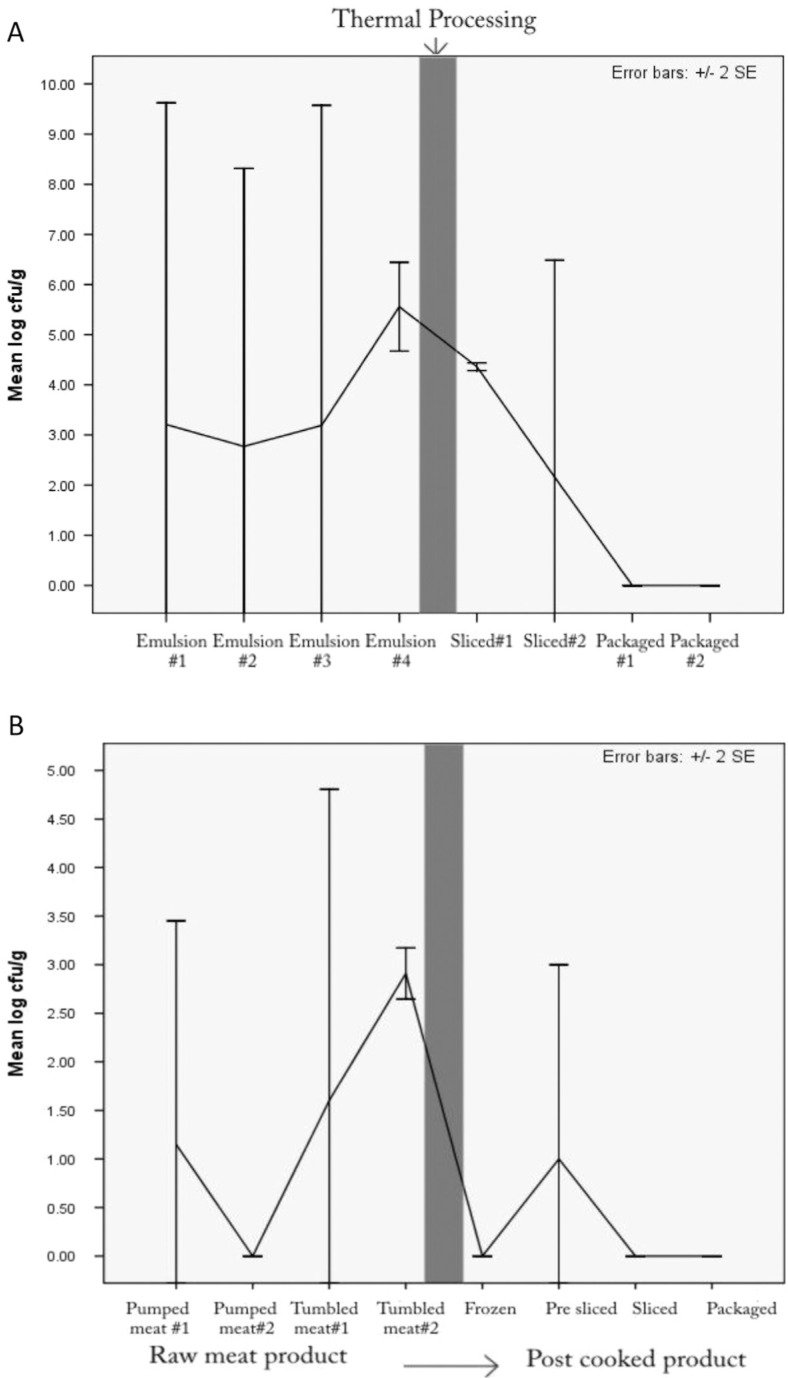
Mean log counts of *S. aureus* in pre- and post-cooking food samples during production of chicken bologna (A) and bacon (B).

*S. aureus* was detected in air, food and environmental samples at an overall prevalence rate of 27.1% (46/170) (Table 2). The organism was isolated from pre-cooked food samples of franks, bologna and bacon (mean = 0.66, 1.98 and 1.95 log_10_CFU/g, respectively) and post-cooked bologna and bacon (1.63 and 0.25 log_10_CFU/g, respectively). Differences between pre- and post-cooked samples were however not statistically significant (*P*>0.05). There was a trend of a general decrease in counts after thermal processing of bologna and bacon (Fig. 3). The frequency of food samples exceeding acceptable limits of *S. aureus* (10^4^ CFU/g) during the manufacture of chicken franks, chicken bologna, and bacon were 11.1% (2/18), 33.3% (6/18), and 20.0% (4/20) for pre-cooked samples, respectively, and 0.0% (0/8), 37.5% (3/8), and 12.5% (1/8) for post-cooked samples, respectively.

Airborne *S. aureus* counts ranged from 0.65 to 2.24 log_10_CFU/ m^3^ (Table 2). However, there were no significant (*P*>0.05) differences in levels between pre- and post-cooking areas during the production of any of the three products (*P*=0.92). *S. aureus* swab counts ranged from 0.83 to 2.42 log_10_CFU/ m^2^ in pre-cooking areas and 0–2.23 log_10_CFU/m2 in post-cooking areas (Table 2). Differences between pre- and post-cooking areas were not statistically significant (*P*>0.05) for all three products. There were *S. aureus* positive swab samples from post-cooking areas including the gloves of packaging employees and the floor of the packaging room during the production of both bologna and bacon (data not shown).

### Prevalence of *E. coli*

The overall prevalence of *E. coli* from all samples was 2.9% (5 of 170). The organism was only found on surfaces and in raw food items in pre-cooking areas (Table 2). *E. coli* was detected in pre-cooked samples of chicken franks and chicken bologna at a prevalence of 5.6% (1/18) and 11.1% (2/18), respectively; however, no *E. coli* was detected in their cooked counterparts or in any food item during bacon production. Mean counts were 0.35 and 0.72 log_10_CFU/g in uncooked franks and bologna samples, respectively. The organism was detected in one pre-flaked raw meat sample during the production of chicken franks and in both raw and flaked meat during the production of bologna. Positive swab samples were from the stuffing equipment during the production of bologna and on the surface of an employee's gloves during pumping of bacon. No *E. coli* O157 strain was detected during the production of franks, bologna and bacon.

### Prevalence of coliforms

Coliform bacteria were detected in food, surfaces and air samples. The overall prevalence of coliforms was 58.8% (100 of 170). Raw meat or uncooked products accounted for all coliform positive samples except for one post-cooked bologna sample and two post-cooked frank samples. The prevalence of coliforms in samples of pre-cooked chicken franks, chicken bologna and bacon was 88.9% (16/18), 88.9% (16/18) and 55.0% (11/20), respectively, and 25% (2/8) and 12.5% (1/8) in post-cooked chicken franks and chicken bologna samples respectively and this difference was significant (*P*<0.05). Average counts were significantly (*P*<0.05) higher in pre-cooked than post-cooked food samples for all three products. Pre-cooked food samples for franks, bologna and bacon had mean coliform counts of 7.09, 5.59 and 3.01 log_10_CFU/g, respectively; whereas post-cooked food samples for franks and bologna were 0.58 and 0.42 log_10_CFU/g, respectively. No coliforms were detected in post-cooked bacon samples.

Coliform levels found in air samples ranged from 0.54 to 1.35 log_10_CFU/m^3^ in pre-cooking areas and from 0.85 to 1.05 log_10_CFU/m^3^ in post-cooking areas during the production of franks and bologna (Table 2). The differences were not statistically significant (*P*>0.05). No airborne coliforms were detected during the production of bacon.

Environmental swabs also showed a trend of lower counts in post-cooking areas (range = 5.8 – 6.7 log_10_CFU/m^2^) when compared to pre-cooking environments (7.06 – 9.17 log_10_CFU/m^2^) for all three products, but the differences were not significant (*P*>0.05) (Table 2).

### Prevalence of *Salmonella spp*

The frequency of isolation of *Salmonella* spp. was 2.9% (5 of 170). The pathogen was only detected in pre-cooked meat (raw or flaked) during the production of chicken franks and chicken bologna at a prevalence of 11.1% (2/18) and 16.7% (3/18), respectively. One isolate from raw meat used for producing chicken franks was serotyped as *Salmonella* Typhimurium and another from flaked meat was identified as *Salmonella* Group C3. Two isolates recovered from pre-processed meat during the production of chicken bologna were identified as *Salmonella* Group C1 and C3; and one isolate identified as Group B was found after flaking.

### Prevalence of *Listeria spp*

The overall frequency of isolation of *Listeria* spp. and *L. monocytogenes* was 14.1% (24 of 170) and 2.4% (4 of 170), respectively. *Listeria* spp. was isolated from food samples and environmental swabs taken during the production of bacon and chicken bologna. *Listeria* spp. was detected in pre-cooked samples of chicken bologna and bacon at a prevalence of 55.6% (10/18) and 50.0% (10/20), respectively. The 10 pre-cooked samples collected during the production of bologna that presented positive for *Listeria* spp. originated from: one raw meat pre-flaking, one post-flaking and all eight samples of uncooked emulsion (raw meat and filler). Four of the emulsion samples were positive for *L. monocytogenes*. One swab from the stuffing equipment was also positive for *Listeria* spp. Ten uncooked food samples collected during the production of bacon were positive for *Listeria* spp.: two liquid cure, one raw pork meat, three meat from the injector before pumping, and four pumped meat being placed in tumblers. Three swab samples were also positive *Listeria* spp: one each from the tumbler and post-cooking slicing equipment on the first visit and one from the pumping equipment on the second visit.

### Temperature and humidity of processing environments

At the processing plant studied, cooking times for chicken franks, bologna, and bacon were 1.5, 6, and 3.5 hours, respectively, and the oven temperature for all products was 78°C. For cold storage, freezers were set at temperatures between −20 and 0°C while, for those which held post-cooked products, temperatures were held between −2 and 0°C. The mean ambient temperature of pre-cooking areas (excluding the oven room) was 20.8°C and for post-cooking areas it was 22.9°C but this difference was not statistically significant (*P*>0.05). However, relative humidity differed significantly (*P*<0.05) between pre-cooking (mean = 77.6%) and post-cooking (mean = 64.1%) areas.

## Discussion

The results of the study show the existence of risks for the microbiological contamination, of finished RTE products processed at the plant investigated. The potential for contamination from air and surfaces appears to be the most important risk factor that can affect the microbiological quality of the processed meats. Some organisms such as *E. coli*, *Salmonella* spp., and *Listeria* spp. were found in raw food samples but not in heat-treated products. This suggests that the heat treatment processes applied to the meats were effective in eliminating pathogenic bacteria. The effectiveness of cooking could also be concluded from the fact that raw meat samples for all products harboured high counts of coliforms and aerobic bacteria, which were detected in substantially lower levels directly after cooking. This is in contrast to an inference of Gibbons et al. (3) that inadequate heat treatments in the same plant may have possibly been the cause for contaminated products.

A major problem observed was the increases in counts of *S. aureus*, aerobic bacteria, and coliforms at stages after heat treatment. The concurrent presence of high bacterial counts in air and on food contact surfaces in the post-processing environment is a clear indication that cross-contamination of post-cooked products may have resulted from these sources. A study by Aycicek et al. (23) reported that processed foods requiring more handling during preparation were found to be more prone to *S. aureus* contamination, and Saide-Albornoz et al. (24) showed increased human handling in a pork processing plant as the main contributor to the linear increase of *S. aureus* from slaughter to fabrication.

The potential for *S. aureus* cross-contamination from employees and the environment to food was also unmistakable, as only when the post-cooking environment harboured *S. aureus* during slicing and packaging operations, were the RTE foods contaminated with this organism. Additionally, *S. aureus* was frequently found on employees’ gloves. Considering that *S. aureus*, which could cause foodborne intoxication, is carried in the nose, throat, hair and skin of humans (25), strict monitoring of GHP's by employees in the processing plant needs to be implemented. Furthermore, *E. coli* was found on the surface of an employee's gloves and this could indicate possible cross-contamination scenarios.

The possibility of cross-contamination of *Listeria* spp. was also evident during the first bacon sampling visit to the plant, where it was found in pumped meat, the tumbler and subsequently the slicing machine used for cutting post cooked items. It is also likely that *Listeria* spp. may have been able to survive in biofilms on the post-cooking equipment and, by extension, the environment where niches could have been formed (8). A similar scenario of recontamination was proposed by a study done in a large meat processing plant in Trinidad (3). However, a more probable explanation for the occurrence of *Listeria* spp. on the equipment may have been due to the movement of employees, equipment and frozen bacon from raw meat areas to the packaging room, thereby resulting in recontamination.

The relatively high prevalence rates and apparent ease of spread displayed by *S. aureus*, *E. coli* and *Listeria* spp. suggest that there is the risk of cross-contamination that may compromise the microbial quality of foods with public health implications. Additionally, considering that the zero tolerance *L. monocytogenes* was found in the plant, workers need to be aware of high-risk activities that may lead to the production of potentially hazardous foods. A study in a chicken processing plant noted that contamination of carcasses with *Listeria* spp. occurred through contaminated surfaces and equipment (17). It is also important to note that *Listeria* spp. is capable of multiplying at refrigeration temperatures (26) and the fact that products tested in this plant are RTE further emphasizes the public health significance of the detection of this pathogen in foods.

The relatively high number of post-cooked franks (25%) and bacon (50%) samples that exceeded recommended limits of aerobic bacteria is suggestive of cross-contamination as well as improper handling and/or storage following cooking. The observed trend of decreasing counts of aerobic bacteria and *S. aureus* after vacuum packaging may have been due to the mechanical evacuation of oxygen, which created an unsuitable environment for growth of microorganisms (27). The only exception to this observation was bacon, where aerobic plate counts continued an increasing trend after heat treatment, up to packaging. It is possible that the associated high fat content of bacon could have protected the bacteria in this product (28). Additionally, some species of bacteria such as *Listeria monocytogenes* are capable of surviving anaerobic conditions, which can lead to greater threats to the integrity of these products (29).

In this study it was found that temperatures in the thawing (mean of 24.6°C), pre-cooking (mean of 22.9°C) and packaging (mean of 20.8°C) areas were not low enough to prevent multiplication of bacteria. A study conducted in the USA showed that, among four meat processing plants, the one with the lowest temperature (7–18°C) had the lowest counts of airborne bacteria (30).

The link between the levels of pathogens in raw meat and downstream processes and products was evident. During the second sampling visit for bologna, *E. coli* was found in the raw meat before and after flaking, and in the stuffing room equipment. Similarly, results from the second visit during bacon production showed that *Listeria* spp. was present in the raw meat before pumping, pumped meat in the tumbler, the tumbling equipment and the post-processing slicing machinery. These findings show that there is a possible linear transfer of bacteria, which could have originated in the raw meat throughout the process chain via employees and, equipment. Similar scenarios have been previously documented for *E. coli* O157 in ground beef patties which was traced back to the surface contamination of raw meat during slaughter (31) and in a cheese factory where machinery was a niche for *Listeria* spp. originating from raw contaminated milk (32).

No areas in the processing plant exceeded relative humidity of 90–95%, the optimum range for the growth of spoilage bacteria such as Pseudomonads (33). There was one exception in the oven room, which housed showers that could have increased the moisture content in the air. Nonetheless, humidity and temperature showed vast fluctuations in almost all processing rooms, which suggest the need for more effective control of these factors.

Aerobic airborne bacteria throughout the plant's processing environment ranged from 20 to 1,050 CFU/m^3^, which was similar to the range of 10–1,310 CFU/m^3^ reported for a Brazilian dairy processing plant where a similar study was conducted (7). The location of both plants in tropical climates may lend an explanation to this occurrence, as mesophilic bacteria prefer higher temperatures afforded by low latitudes. The effect of warm temperatures on microbial load of food processing environments was also documented in a poultry processing plant in the US where bacterial counts were higher during warmer summer months when compared to winter months (30).

Air samples taken during the course of the study showed that there were generally lower levels of coliforms, *S. aureus* and aerobic bacteria in the post-cooking room when compared with the raw meat areas. This indicates that measures implemented, such as the use of filters in air conditioning units and directing the airflow from the packaging room to raw meat areas, were preventing some bacteria from entering the area but were not totally effective. Such equipment responsible for managing aerosol particles should therefore be closely monitored and regulated (7). A similar pattern was documented in the US plant, which was attributed to an exemplary airflow from clean packaged areas to ‘dirty’ raw meat areas (30).

All water samples tested were negative for coliforms; however, three samples from the post-processing area contained levels of aerobic bacteria which exceeded EPA guidelines of 500 CFU/ml for drinking water, as recommended by the USDA Food Safety Inspection Service for processing of foods (22). This indicates that water used in the plant studied may contribute to the contamination of products and that manufacturers must make an effort to improve the quality of water used during food processing, particularly in post-cooking processes. Rinse wash analysis of packaging materials showed that packages used did not pose a significant contamination risk.

*Salmonella* spp. was isolated from raw chicken-based meats, a finding in agreement with previous studies on poultry, which have asserted this meat as a carrier of this pathogen (34, 35). It was significant that post-cooked meats and environments were all negative for *Salmonella* spp. However, deli meats have been previously associated with human cases of salmonellosis (36).

It was also noticed that during bacon production, after the liquid cure was pumped into the uncooked pork, levels of coliforms significantly decreased. This could be attributed to the inhibitory effect of spices found in the cure on coliforms. Studies done on compounds derived from spices and herbs have also documented their inhibitory effect on bacterial species such as *Clostridium*, *Salmonella*, and *Escherichia* (37, 38).

## Conclusion

There is a relatively high level of risk of microbiological contamination of products manufactured at the food plant investigated. It seems that re-contamination may be the most plausible explanation for products presenting with unacceptable levels of bacteria. This could be largely attributed to inadequate GHPs, GMPs, and the lack of separation between cooked and uncooked products, as well as equipment in pre- and post-cooking areas. The use of wooden pallets, dirty walls and floors as well as the storage of raw materials near the ground and garbage may have also contributed to contamination. Re-modeling of the plant with an appropriate design is necessary to allow for proper traffic flow, and with the implementation of the aforementioned recommendations, the quality and safety of goods produced by this plant could be improved substantially.
